# Predicting the risk of Lyme borreliosis after a tick bite, using a structural equation model

**DOI:** 10.1371/journal.pone.0181807

**Published:** 2017-07-24

**Authors:** Agnetha Hofhuis, Jan van de Kassteele, Hein Sprong, Cees C. van den Wijngaard, Margriet G. Harms, Manoj Fonville, Arieke Docters van Leeuwen, Mariana Simões, Wilfrid van Pelt

**Affiliations:** 1 Centre for Infectious Disease Control Netherlands, National Institute for Public Health and the Environment, Bilthoven, the Netherlands; 2 Department of Statistics, Informatics and Mathematical Modeling, National Institute for Public Health and the Environment, Bilthoven, the Netherlands; University of Maryland, College Park, UNITED STATES

## Abstract

**Background:**

Understanding and quantification of the risk of Lyme borreliosis after a tick bite can aid development of prevention strategies against Lyme borreliosis.

**Methods:**

We used 3,525 single tick bite reports from three large prospective studies on the transmission risk of tick-borne pathogens to humans, with 50 reports of Lyme borreliosis during the follow-up period, among 1,973 reports with known outcome. A structural equation model was applied to estimate the risk of Lyme borreliosis after a tick bite, and quantify the influence of: developmental stage of the tick, detection of *Borrelia burgdorferi* s.l. DNA in the tick by PCR, tick engorgement, patient-estimated duration of tick attachment, and patient age.

**Results:**

The overall risk of developing Lyme borreliosis after a tick bite was 2.6% (95%CI 1.4–5.1).

The risk increased with:

- Tick engorgement: 1.4% (95%CI 0.7%-2.3%) for low engorgement to 5.5% (95%CI 2.8%-9.2%) for substantially engorged ticks;
- Rising patient-estimated tick attachment duration: 2.0% (95%CI 1.3%-2.8%) after <12 hours, to 5.2% (95%CI 3.0%-8.9%) after ≥4 days;
- Detection of *Borrelia burgdorferi* s.l. DNA in ticks: 6.7% (95%CI 3.6%-13.5%), versus 1.4% (95%CI 0.7%-2.9%) when ticks tested negative.

The highest observed risk of Lyme borreliosis was 14.4% (95%CI 6.8%-24.6%) after one tick bite of a substantially engorged tick that tested positive for *Borrelia burgdorferi* s.l. DNA, which corresponds to one new case of Lyme borreliosis per 7 (95%CI 4–15) of such tick bites.

**Conclusions:**

An individual's risk of Lyme borreliosis after a tick bite can be predicted with tick engorgement, patient-estimated duration of tick attachment, and detection of *Borrelia burgdorferi* s.l. DNA in the tick.

## Introduction

Lyme borreliosis is a tick-borne disease caused by bacteria of the *Borrelia burgdorferi* sensu lato group (hereafter referred to as Borrelia). The most common clinical manifestation of Lyme borreliosis is erythema migrans, an expanding skin lesion indicating early localized infection at the site of the tick bite. Late and more serious Lyme borreliosis can present with skin, neurological, musculoskeletal and cardiac manifestations[1]. The incidence of Lyme borreliosis has increased markedly in several regions of Europe[2]. To aid development of prevention strategies against Lyme borreliosis, understanding and quantification of the risk of developing Lyme borreliosis after a tick bite are required.

The risk of infection with Borrelia after a tick bite depends on several factors, one of these being the prevalence of Borrelia in ticks. Ticks have four life stages: eggs, larva, nymph and adult. After hatching from the eggs, ticks need a blood meal from a vertebrate before dropping off to moult to the next stage or to lay eggs in the case of an adult female. During a blood meal from an infected animal, ticks can become infected with Borrelia, and during the subsequent blood meals the bacteria can be transmitted to new hosts. [3] The tick infection rate rises with its developmental stage. Among field collected host-seeking ticks, less than 1% of larvae are infected, about 10% to 30% of the nymph, and 15% to 40% of adults[4–6]. Another factor is the transmission of Borrelia from ticks to humans, which increases with the duration of the tick’s blood meal, and can be quantified as patient-estimated duration of the tick bite or as degree of engorgement of the tick. As described for the North American vector *Ixodes scapularis*, detectable transmission of *Borrelia burgdorferi* sensu stricto requires attachment to the host for at least 24 hours in animal experiments [7, 8]. In Europe, Lyme borreliosis is transmitted by *Ixodes ricinus* and caused mainly by other species of *Borrelia* such as *B*. *afzelii* and *B*. *garini*. Transmission of *B*. *afzelii* has been reported within the first 24 hours of *Ixodes ricinus* attachment in an animal experiment[9, 10]. And tick attachment durations shorter than 24 hours have been reported by patients with Lyme borreliosis in human observational studies in Europe.[11–14]

The recommended standard practice after tick removal is watchful waiting; to monitor the skin for development of erythema migrans or other clinical symptoms indicative of Lyme borreliosis. As an alternative, the medical guidelines in the United States 2006[15] and the Netherlands since 2013[16] mention prophylactic antibiotic treatment of tick bites with a single 200-mg dose of doxycycline administered within 72 hours after tick removal. Based on a study from the United States prophylactic treatment is estimated to be 91% effective (95%CI 42%–100%), and that about fifty people bitten by a tick would need treatment to prevent one case of Lyme borreliosis.[17] We aim to explore to what extent the number needed to treat could be reduced through identification of patients with high risk of developing Lyme borreliosis. In the current article we model the risk of Lyme borreliosis after a tick bite, and we investigate the effect of possible predictors such as the developmental stage of the tick, tick engorgement, detection of Borrelia DNA in the tick, patient-estimated duration of tick attachment, and patient age. The estimated risks for Lyme borreliosis from our prediction model might be useful in clinical practice to identify persons with a higher risk of developing Lyme borreliosis.

## Methods

### Data description of three nationwide prospective studies

To obtain sufficient numbers of subjects for estimation of the effect of the predictors of the risk of Lyme borreliosis after a tick bite, data of three nationwide prospective studies on the transmission risk of tick-borne pathogens to humans in the Netherlands between 2007 and 2013 were combined. It was assumed that the effect of the predictors on risk of Lyme borreliosis did not change between 2007 and 2013. S1 Table provides summary measures of the combined dataset, which contains 3,525 single tick bite reports, 50 reports of Lyme borreliosis during the follow-up period, and 1,552 reports with unknown outcome due to loss to follow-up. S1 File contains the full dataset. Only participants who did not report other tick bites during the six weeks before or after enrollment, and participants who did not report prophylactic antibiotic treatment for a tick bite at enrolment were included. Study TR1213 was a web-based national survey on www.tekenradar.nl, through which civilians reported 3,191 tick bites between March 2012 and March 2013, with 42 reports of Lyme borreliosis within three months follow-up. [18, 19] Study GP0708 was a nationwide prospective study among 260 patients with tick bites who consulted one of 307 enrolling general practitioners in 2007 and 2008, with 3 reports of Lyme borreliosis within three months follow-up[12]. Study EP0911 was a prospective study performed among 244 patients with tick bites who visited one of fourteen medical emergency posts for consultation of a general practitioner outside office hours from 2009 to 2011, with 5 reports of Lyme borreliosis within two months follow-up.

All study participants were asked to fill out questionnaires at enrollment and at follow-up, including questions on duration of tick attachment in the skin, the number of tick bites, and development of Lyme borreliosis. In TR1213 and GP0708, patient reported development of Lyme borreliosis was confirmed through an additional questionnaire sent to their general practitioner. Our outcome measure Lyme borreliosis within three months after a tick bite was categorized as “physician-confirmed erythema migrans” or “physician-confirmed disseminated Lyme borreliosis” when patient-reported Lyme borreliosis was confirmed by the general practitioner through that additional questionnaire. In EP0911, and for participants of TR1213 and GP0708 whose general practitioner did not respond to the questionnaire, patient-reported erythema migrans combined with prescribed antibiotics was categorized as “probable erythema migrans”. Our outcome measure Lyme borreliosis after a tick bite contained 29 physician-confirmed erythema migrans, and 21 probable erythema migrans with antibiotic treatment.

Ticks removed from the skin were submitted to our study laboratory at the National Institute of Public Health and the Environment of the Netherlands through regular mail, and examined under a microscope to determine tick species, developmental stage, gender, using standard keys [20]. Trained laboratory employees categorized the degree of engorgement of the tick as low, moderate, or substantial engorgement through visual inspection. No other tick species than *Ixodes ricinus* were identified in GP0708.[12] The species of 187 ticks in EP0911 were not recorded during visual inspection by our trained laboratory employees for tick stage and engorgement. During cleaning of the TR1213 data, one *Dermacentor marginatus* tick, one *Ixodes hexagonus*, and two *Dermacentor reticulatis* ticks had been excluded from the dataset. Total DNA was extracted from the collected ticks, to test the tick lysates for DNA of Borrelia. A duplex quantitative (Q)PCR using fragments of the outer membrane protein A (OspA) gene and the flagellin B (FlaB) gene as targets[21] was applied to tick lysates from TR1213 and from EP0911 collected in 2011. A real-time QPCR on the OspA gene[22] and by reverse line blotting (RLB)[23, 24] was applied to tick lysates from GP0708 and from EP0911 collected in 2009. Tick lysates from EP0911 ticks collected in 2010 were analyzed by RLB[23, 24].

In GP0708 all participants (or their parents / guardians) gave written informed consent for analysis of a paired blood sample (blood sample data were not used for the current manuscript), and the study protocol of GP0708 was approved by the medical ethics review committee of the University Medical Centre in Utrecht, the Netherlands (number 07-032/K). EP0911 and TR1213 did not involve burdensome procedures (e.g. collection of blood samples). The Medical Ethics Review Committee UMC Utrecht declared that the Medical Research Involving Human Subjects Act does not apply to TR1213 (number 15-734/C). The data of these three studies were anonymized before data analysis.

### Structural equation model

We aimed to quantify the risk of Lyme borreliosis after a tick bite, and to quantify the influence of characteristics of the tick (bite) and the person’s age. Some of these characteristics interact with each other, so we applied structural equation modeling where all (causal) relations between variables are combined in one model, which allows for natural sequence of causation, as opposed to for instance multivariable regression analysis.

**Fig 1:**
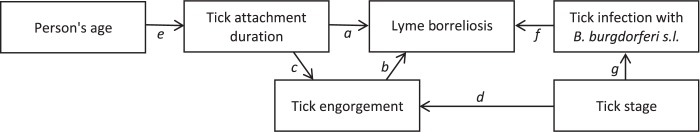
Variables [blocks] and assumed (causal) relations [arrows a to g] between predictors of Lyme borreliosis after a tick bite in the structural equation model.

Fig 1 shows the structure of our equation model, with our outcome measure Lyme borreliosis as the central variable, and the variables about the person (age) and the tick (the tick’s developmental stage, engorgement, attachment duration, and whether DNA of Borrelia is detected in the tick). The arrows ‘a’ to ‘g’ between these variables in Fig 1 depict (causal) relations between variables. We assumed that the risk of Lyme borreliosis increases with the patient-reported tick attachment duration (arrow a) or with tick engorgement (arrow b)[9, 10]. Additionally, we assumed that engorgement increases with rising patient-reported tick attachment duration (arrow c)[25], and that engorgement may differ per developmental stage of the tick (arrow d). We further assumed that older study participants report longer tick attachment durations than younger participants (arrow e)[26, 27]. We assumed that the risk of Lyme borreliosis is higher when DNA of Borrelia is detected in the tick (arrow f) [12, 13, 28, 29], and that the probability of a tick carrying Borrelia is associated with the developmental stage of the tick (arrow g) [4–6]. Through explorative analyses of each variable and associations between the variables, several conventional statistical models (e.g. time-to-event model, proportional odds model) were applied for specific parts of the model, and assembled into one structural equation model. For each sub-model we checked how well the associations were described and the outcome was predicted. The cumulative risk of Lyme borreliosis is described by a proportional hazards model with a Weibull baseline hazard function. The baseline hazard depends on the tick's engorgement and the patient-estimated tick attachment duration. The complementary log-log of the risk of Lyme borreliosis is a linear function of the log tick attachment duration. The tick infection with Borrelia enters the equation via a proportional hazards term. Explorative analyses of our data indicated that the tick attachment duration could be well described by a log-Normal distribution that also allows for durations that were reported as an interval. Explorative analyses of our data also indicated that the mean log attachment duration is a linear function of age. The participant's age (reported as integers) is described by a categorical distribution (Fig 1). The tick infection with Borrelia is described by a Bernoulli distribution, having one parameter, the prevalence of Borrelia in ticks in our dataset. The logit of the probability of tick infection with Borrelia is a function of the tick's developmental stage, which is described by a categorical distribution (larva, nymph, adult). The probability of each engorgement class is described by a proportional odds logistic regression model, where the logit of the cumulative probabilities is a cubic function of the log attachment duration, with a tick stage specific intercept, and one cut point to describe the log odds for transition from engorgement class 'moderate' to 'substantial'.

### Data preparation for combined analysis and data imputation

We used a Bayesian approach, using Markov Chain Monte Carlo, to estimate the parameters. The advantage of this approach is that our complex structural equation model could be formulated in a straightforward manner. Besides, missing predictive variables were automatically imputed and it also naturally coped with uncertainties in the combined dataset.

We assumed that the missing value mechanism for the predictive variables was “missing completely at random” (MCAR), meaning that the probability of a missing value (in tick stage, engorgement, tick infection, tick attachment duration, or age) was unrelated to any other variables. Missing values were sampled from the probability distributions that we assigned to these variables as described in de section “Structural equation model”. We put non-informative priors on all parameters and generated 10,000 posterior samples for each parameter. Convergence was checked visually. All data pre- and post-processing were done in R. Computations were done in JAGS[30] (see JAGS model code in S2 Table). Participants with unknown outcome “Lyme borreliosis after a tick bite” due to loss to follow-up within three months after enrollment (*n* = 1,552) were omitted from estimation of the risk of Lyme borreliosis. However, their other available data (tick stage, engorgement, tick infection, tick attachment duration, age) were used for parameter estimation to make inference about missing data. The patient-reported duration of tick attachment in the skin was available as real valued data (hours) as well as interval censored data, depending on the study. For the TR1213 study, the duration was only provided in intervals (0–12 hours, 12–24 hours, and >24 hours). For the GP0708 study, the duration was provided as real valued data (hours) as well as in intervals (<24 hours, >24 hours). For the EP0911 study, the duration was provided only in intervals (0–12 hours, 12–24 hours, >24 hours). For combined analysis, these patient-reported attachment durations were handled as follows: if the duration was reported as a real value (in hours), then this value was taken as is. If the duration was reported as an interval, the lower- and upper bound were specified in hours and the duration itself was set to a missing value. If no attachment duration was reported, the lower bound was set to 1 hour and the upper bound was set to 240 hours. The missing duration values were subsequently imputed during the estimation procedure by sampling from the log-Normal distribution with the specified lower- and upper boundaries. Explorative analyses showed that the patient-reported hours of tick attachment centered toward whole days (24, 48, 72 or 96 hours) for the TR1213 study, which occurred much less in GP0708 and EP0911. We therefore widened the interval with 25% (lower and upper boundary) around the patient-reported hours of tick attachment in TR1213, and with 1% for the GP0708 and EP0911 studies.

## Results

### Characteristics of participants and their tick (bite)

**Fig 2:**
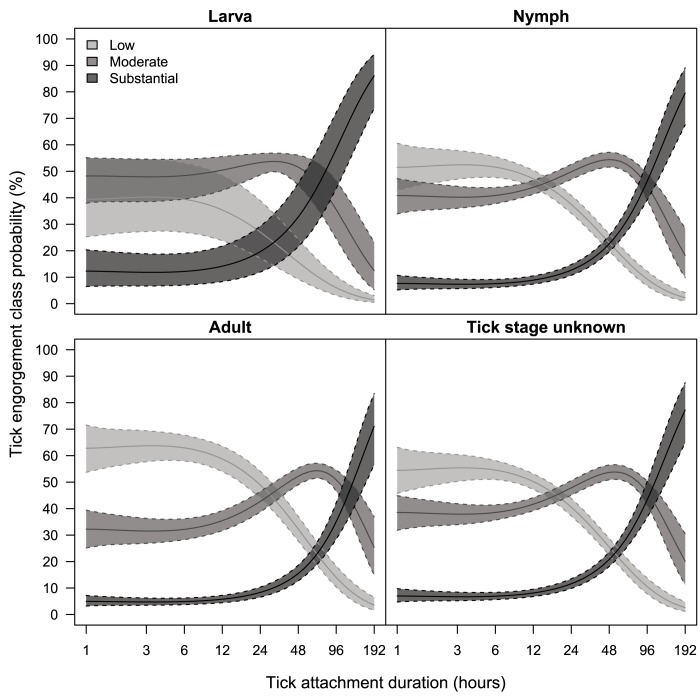
Probability of tick engorgement classes as a function of patient-estimated duration of tick attachment, per tick stage (ignoring tick infection with *Borrelia burgdorferi* s.l. DNA, and age of the participant). The solid line represents the mean, the dotted lines the 95% credible interval.

Table 1 shows the occurrence in our dataset of developmental stage of the tick, categories of tick engorgement, categories of patient-estimated duration of tick attachment, detection of Borrelia DNA in ticks, and age of the participant. Most ticks removed from our study participants were nymphs (68%), or adult ticks (29%), and a small proportion of the ticks were larvae (3%). The duration of tick attachment, as estimated by the participants, ranged from 0.2 hours to 16 days with a median of 13.5 hours, and a mean of 27 hours (95%CI: 1.3–135.0). Tick attachment duration shorter than 12 hours were reported by 46% of the participants, and tick attachment durations longer than four days were rarely reported (4.8%). Per every 10-year increase of a person’s age, the mean duration of tick attachment slightly increased with 1.06 hours (95%CI 1.04–1.09). Of the ticks in this combined dataset, 14% were substantially engorged. Among these participants with a substantially engorged tick, the mean tick attachment duration was 69.2 hours (95%CI 1.9–323.3). Most ticks were categorized as moderate (43%) or low (43%) engorgement, and the mean tick attachment durations among these participants were 25.4 hours (95%CI 1.4–105.2) and 15.1 hours (95%CI 1.4–69.8) respectively. Fig 2 shows how the degrees of tick engorgement correspond to patient-estimated duration of tick attachment per developmental stage of the tick. The overall probability of Borrelia DNA detection in ticks removed from humans was 21.6% (95%CI 19.9%–23.5%) (Table 1). The probability of DNA detection rose with the developmental stage, from 7.4% for larvae (95%CI 2.2%–15.7%), to 19.2% (95%CI 17.1%–21.3%) for nymphs, and 28.7% (95%CI 25.1%–32.4%) for adult ticks. Compared to larvae, adult ticks removed from humans were five times more likely to test positive for Borrelia DNA (hazard ratio 5.0, 95%CI 1.8–13.2) with statistical significance, as well as nymphs compared to larvae (hazard ratio 3.4, 95%CI 1.2–9.1).

**Table 1:** Probability of developing Lyme borreliosis after one* tick bite, with predictors: Developmental stage of the tick, tick engorgement, tick infection with *Borrelia burgdorferi* s.l. DNA, patient-estimated duration of tick attachment, and age of the participant. See supporting information S3 Table for more risk estimates of these combined predictors.

	Risk%	(95% CI)	Risk%	(95% CI)	Risk%	(95% CI)	Risk%		(95% CI)
**Tick stage** *[% occurrence in dataset]* Risk% (95% CI), ignoring^‡^ tick infection with *Borrelia burgdorferi* s.l. DNA, tick engorgement, tick attachment duration, and age.									
	**Ignoring**^‡^ **tick stage**		**Larva** *[2*.*8%]*		**Nymph** *[68*.*2%]*		**Adult** *[29*.*1%]*		
	2.6%	(1.4%–5.1%)	2.1%	(1.1%–4.1%)	2.5%	(1.4%–4.9%)	2.7%	(1.5%–5.5%)	
**Engorgement** *[% occurrence in dataset]* Risk% (95% CI), ignoring^‡^ tick infection with *Borrelia burgdorferi* s.l. DNA, duration of tick attachment, and age.									
Low *[43*.*1%]*	1.4%	(0.7%–2.3%)	1.0%	(0.4%–1.7%)	1.3%	(0.6%–2.2%)	1.6%	(0.8%–2.7%)	
Moderate *[43*.*0%]*	2.8%	(1.6%–4.2%)	2.0%	(1.1%–3.2%)	2.7%	(1.6%–4.0%)	3.2%	(1.9%–4.8%)	
Substantial *[13*.*9%]*	5.5%	(2.8%–9.2%)	3.9%	(1.8%–6.8%)	5.3%	(2.6%–8.7%)	6.4%	(3.2%–10.6%)	
**Patient-estimated duration of tick attachment** *[% occurrence in dataset]* Risk% (95% CI), ignoring^‡^ tick infection with *Borrelia burgdorferi* s.l. DNA, engorgement, and age.									
<12 hours *[46*.*1%]*	2.0%	(1.3%–2.8%)	1.7%	(1.0%–2.5%)	2.0%	(1.3%–2.7%)	2.2%	(1.4%–3.0%)	
12 to 24 hours *[22*.*8%]*	2.4%	(1.7%–3.1%)	2.0%	(1.3%–2.9%)	2.3%	(1.7%–3.1%)	2.6%	(1.8%–3.4%)	
24 to 48 hours *[17*.*2%]*	2.8%	(2.1%–3.8%)	2.3%	(1.5%–3.5%)	2.8%	(2.0%–3.7%)	3.0%	(2.2%–4.1%)	
2 to 4 days *[9*.*1%]*	3.6%	(2.5%–5.2%)	2.9%	(1.8%–4.5%)	3.5%	(2.4%–5.1%)	3.9%	(2.6%–5.6%)	
≥ 4 days *[4*.*8%]*	5.2%	(3.0%–8.9%)	3.9%	(2.1%–7.0%)	5.0%	(2.9%–8.6%)	5.7%	(3.3%–9.9%)	
**Tick infection with *Borrelia burgdorferi* s.l. DNA** *[% occurrence in dataset]* Risk% (95% CI), ignoring^‡^ engorgement, duration of tick attachment, and age.									
Negative *[78*.*4%]*	1.4%	(0.7%–2.9%)	1.6%	(0.8%–3.2%)	1.4%	(0.7%–3.0%)	1.3%	(0.6%–2.7%)	
Positive *[21*.*6%]*	6.7%	(3.6%–13.5%)	7.7%	(4.0%–14.9%)	6.9%	(3.7%–13.7%)	6.3%	(3.3%–12.6%)	
**Age of participant** *[% occurrence in the population of the Netherlands]* Risk% (95% CI), ignoring^‡^ tick infection with *Borrelia burgdorferi* s.l. DNA, engorgement, and duration of tick attachment.									
<20 years *[22*.*7%]*	2.4%	(1.4%–4.6%)	2.0%	(1.0%–3.7%)	2.4%	(1.4%–4.5%)	2.6%	(1.5%–5.0%)	
20 to 40 years *[24*.*5%]*	2.5%	(1.4%–4.9%)	2.0%	(1.1%–4.0%)	2.5%	(1.4%–4.8%)	2.7%	(1.5%–5.3%)	
40 to 70 years *[41*.*1%]*	2.6%	(1.5%–5.2%)	2.1%	(1.1%–4.2%)	2.5%	(1.4%–5.1%)	2.8%	(1.5%–5.6%)	
≥ 70 years *[11*.*8%]*	2.7%	(1.5%–5.6%)	2.2%	(1.1%–4.4%)	2.6%	(1.4%–5.4%)	2.9%	(1.6%–6.1%)	

95% CI: 95% credible interval based on 2.5% and 97.5% quantiles.

*For multiple independent tick bites these risk percentages can be combined: *P*_*total*_ = 1–(1—*P*_*tick1*_) x (1—*P*_*tick2*_) x … x (1—*P*_*tickN*_).

‡Marginal probabilities were calculated per predictor, averaged over all the other predictive variables in the model.

### Quantification of predictors of the risk of Lyme borreliosis

The overall risk of developing Lyme borreliosis after a tick bite was 2.6% (95%CI 1.4%–5.1%). Here, “overall” means averaged over all predictive variables in the model (ignoring tick infection with Borrelia DNA, tick engorgement, tick stage, tick attachment duration, and age).

Table 1 and S3 Table show the probabilities for developing Lyme borreliosis after a tick bite, for each predictor in our structural equation model: developmental stage of the tick, tick engorgement, detection of Borrelia DNA in the tick, patient-estimated duration of tick attachment, and age of the participant.

**Fig 3:**
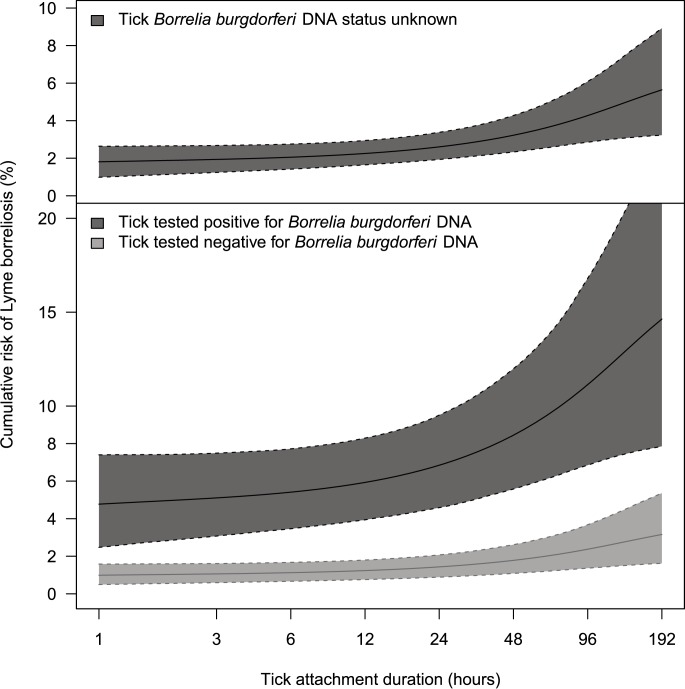
Probability of developing Lyme borreliosis after a single tick bite, as a function of patient-estimated duration of tick attachment. The solid line represents the mean, the dotted lines the 95% credible interval. Also see Table 1 and S3 Table. **Upper graph: ignoring all other variables in our model. Lower graph: stratified for tick infection with *Borrelia burgdorferi* s.l. DNA, tested by PCR. Ignoring tick stage, engorgement, and age of the participant.**

Fig 3 shows how the risk of Lyme borreliosis increases with patient-estimated tick attachment duration; rising from 2.0% (95%CI 1.3%–2.8%) during the first 12 hours of tick attachment to 5.2% (95%CI 3.0%–8.9%) after four days (upper graph in Fig 3, and S3 Table). These risk estimates for patient-estimated tick attachment duration “<12 hours” and “≥4 days” differ with statistical significance, as their 95%CI do not overlap. Ticks that tested positive for Borrelia posed a five times higher hazard (statistically significant hazard ratio 5.2, 95%CI 2.5–7.5), compared to ticks that tested negative (6.7% (95%CI 3.6%–13.5%) versus 1.4% (95%CI 0.7%–2.9%) risk of developing Lyme borreliosis). Hence, when a tick tested positive for Borrelia, the risk of Lyme borreliosis increased from 5.4% (95%CI 3.2%–7.8%) during the first 12 hours of tick attachment, to 13.5% (95%CI 7.4%–23.5%) after four days (lower graph in Fig 3, and S3 Table). Per degree of tick engorgement, the risk of Lyme borreliosis increased with statistical significance from 1.4% (95%CI 0.7%–2.3%) for low engorgement to 5.5% (95%CI 2.8%–9.2%) for substantially engorged ticks (Table 1). The difference between risk estimates per developmental stage of the tick and for age of the participant were not statistically significant (Table 1).

### Estimating an individual’s risk of Lyme borreliosis after a tick bite

An individual’s risk of Lyme borreliosis after a tick bite can be estimated with the probabilities in Table 1 and S3 Table. To illustrate; when an adult tick is removed from the skin after a patient-estimated tick attachment duration of two to four days, the mean risk of developing Lyme borreliosis is estimated at 3.9% (95%CI 1.8%–6.8%) (Table 1, S3 Table). At the overall 2.6% risk of Lyme borreliosis after a tick bite, one new case of Lyme borreliosis would develop per 38 (95%CI 20–71) tick bites. The highest risk of Lyme borreliosis after a tick bite we identified with our model, was 14.4% (95%CI 6.8%-24.6%) after a tick bite of a substantially engorged tick that tested positive for Borrelia (see S3 Table), which corresponds to one new case of Lyme borreliosis per 7 (95%CI 4–15) tick bites. The occurrence of substantially engorged ticks that tested positive for Borrelia was 2.9% in our dataset. Without tick testing for DNA of Borrelia, the highest observed risk of Lyme borreliosis was 6.4% (95%CI 3.2%–10.6%) after a tick bite of a substantially engorged adult tick (see S3 Table), which corresponds to one new case of Lyme borreliosis per 16 (95%CI 9–31) tick bites. The occurrence of substantially engorged adult ticks was 3.1% in our dataset.

For multiple independent tick bites the probabilities of developing Lyme borreliosis after single tick bites can be combined: *P*_*total*_ = 1–(1—*P*_*tick1*_) x (1—*P*_*tick2*_) x … x (1—*P*_*tickN*_). For example, if two substantially engorged nymphs (both mean risk 5.3%; Table 1, S3 Table) and one moderately engorged adult tick (mean risk 3.2%; Table 1, S3 Table) are removed from a person’s skin, the cumulative risk of developing Lyme borreliosis would be: 1–(1–0.053) x (1–0.053) x (1–0.032) = 0.132 = 13.2%; or if four nymphs are removed from the skin after a patient-estimated tick attachment duration of 24 to 48 hours (each mean risk 2.8%; Table 1, S3 Table), the cumulative risk of developing Lyme borreliosis would be: 1–(1–0.028) x (1–0.028) x (1–0.028) x (1–0.028) = 0.1074 = 10.7%.

## Discussion

We show that an individual’s risk of Lyme borreliosis after a tick bite can be estimated with the predictors tick engorgement, detection of DNA of Borrelia in the tick and patient-estimated duration of tick attachment. We observed a 2.6% overall risk of Lyme borreliosis after a tick bite, which is in line with the range of risk estimates of 0.8% to 5.2% that were reported from prospective studies in Sweden, Finland and Switzerland.[13, 28, 29, 31] The relative proportions of Lyme borreliosis diagnoses in the Netherlands have recently been estimated at 95% erythema migrans, and 5% disseminated Lyme borreliosis.[32] Based on these proportions, the currently observed risk of 26 erythema migrans per 1000 recognized tick bites would translate into 1.5 (approximately one or two) additional diagnoses of disseminated Lyme borreliosis per 1000 recognized tick bites. Based on the above mentioned proportions, approximately three diagnoses of disseminated Lyme borreliosis would be expected in our dataset of 1973 tick bites with follow-up. We did not observe disseminated Lyme borreliosis diagnoses in the current study, which may be coincidence (due to insufficient numbers), or possibly because disseminated Lyme borreliosis is less likely to develop within our three month follow-up period. Not having observed development of disseminated Lyme borreliosis in the current study, and knowing that erythema migrans is often not observed preceding to disseminated Lyme borreliosis [33–36], we may have slightly underestimated the risk of developing Lyme borreliosis after a tick bite.

Detection of Borrelia DNA in the tick was our strongest and statistically significant predictor for the risk of developing Lyme borreliosis. Elevated risk estimates for individuals with Borrelia-positive ticks have been reported from previous studies, although not with statistically significant risk difference, which in some studies may be due to insufficient numbers, and in some studies statistical significance had not been investigated for detection of Borrelia DNA in the tick [13, 28, 29, 31]. Counterintuitively, development of Lyme borreliosis was also observed after bites by ticks in which Borrelia DNA was not detected (1.4% risk of developing Lyme borreliosis, 95%CI 0.7%–2.9%). This could be due to other unnoticed tick bites, as it is estimated that around 30%-60% of all tick bites go unnoticed [11, 12, 37], or due to the fact that the sensitivity to detect Borrelia DNA in ticks is less than 100%. Unnoticed extra tick bites in our dataset may have caused a slight overestimation of the risk of Lyme borreliosis after one tick bite.

However, for the clinical relevance of our estimates for a single tick bite it does not matter how many unnoticed tick bites occurred simultaneously, since our study participants most likely have a similar chance of unnoticed tick bites as patients reporting tick bites in daily clinical practice. Before exclusion from the dataset for the current analyses, around 5% of the participants submitted more than one tick that was removed from the skin (17/299 in GP0708, 9/246 in EP0911). We assume that the prevalence of simultaneous unnoticed extra tick bites in our dataset will be much lower than the 30% to 60% unnoticed tick bites among patients with Lyme borreliosis, because detection of one tick bite will most probably incite a thorough further inspection for ticks on the body. Therefore we hypothesize that no more than 1.5% (a third of 5%) of the tick bite participants in our dataset may have had unnoticed extra simultaneous tick bites. In absence of assays for testing of ticks for Borrelia DNA, identification of the tick’s developmental stage can be informative for individual risk assessment, as the infection rate with Borrelia rises with the developmental stage of ticks. However, the risk differences between developmental stages of the tick were not statistically significant, and specifically the risk estimates for larval tick bites should be considered with caution, as only 53 of our observations were larval tick bites, none of which developed erythema migrans. Among field collected host-seeking larvae, prevalence rates of *B*. *burgdorferi* DNA are typically around 1% [3–5], as transovarial (vertical) transmission of *B*. *burgdorferi* sensu lato is ineffective in *Ixodes* ticks[38]. However, like several other studies[39], we detected Borrelia DNA in (7.4% of 53) larvae removed from study participants. A possible explanation for this observation may be the blood meal that ticks removed from study participants had, as the spirochetes multiply rapidly in feeding infected ticks, increasing the spirochete density and thus the chance of detecting Borrelia DNA by PCR.[40]

With longer durations of the tick’s blood meal, the risk of Lyme borreliosis after a tick bite increased significantly, either measured as patient-estimated tick attachment duration, or measured as tick engorgement. Elevated risk estimates for individuals with longer durations of the ticks blood meal have been reported from previous studies, although rarely with statistically significant risk difference [13, 28, 29, 31]. Both measures of the duration of the tick’s blood meal have their specific limitations. Tick engorgement provides poor discrimination, specifically for tick attachment durations below 24 hours, as observed in experimental studies.[14] The reliability and accuracy of patient-estimated tick attachment time is difficult to assess. When tick bitten persons are asked to estimate the duration of the tick’s blood meal, their answer will most likely be based on the most plausible moment of tick exposure and the moment that the tick bite was identified. Wilhelmsson *et al*.[26] compared tick engorgement (based on scutal and coxal indices calculated into hours of tick-feeding) to patient-estimated durations of tick attachment. The majority of tick-bitten persons underestimated the duration of tick attachment, with the strongest underestimation among participants with tick attachment durations longer than two days.[26] Despite these difficulties in assessment of the duration of the tick’s blood meal, we observed an acceptable correlation between tick engorgement and patient-estimated tick attachment duration (Fig 2) in our dataset.

Future refinement of this model could be aimed at investigation of the influence of the species of Borrelia in ticks. In our dataset, the detected DNA of Borrelia in ticks were not analyzed further for identification of the species. Another possibly interesting predictor to add to the model could be the load of Borrelia in the tick, as Wang *et al*.[41] speculated that low numbers (≤ 300) cells of Borrelia found in unfed field-collected ticks may represent a transmission threshold. One may speculate that heavily infected ticks may pose a higher risk of Borrelia transmission, although, in an earlier observational study by Wilhelmsson *et al* [13], the Borrelial load in ticks did not differ significantly between those removed by participants who later seroconverted, and those removed by participants who did not seroconvert. Gender and skin color could possibly be interesting to add to the predictors in the model, as physiological (e.g. more hairy skin or darker skin color resulting in a longer time to detection of a tick on the skin), behavioral, or immunological aspects may influence differences in time to detection of a tick on the skin. Wilhelmsson *et al* reported that men took more time to detect and remove a skin-attached tick, compared to women.[26] Lastly, we aimed to model the risk of Lyme borreliosis after an *Ixodes ricinus* tick bite, although the species of 187 ticks from EP0911 were not recorded. A bite from another tick species than *Ixodes ricinus*, which rarely occurs in the Netherlands, would result in a different risk of Lyme borreliosis. The uncleaned TR1213 dataset contained 0.1% (4/2956) other tick species than *Ixodes ricinus*.

As an alternative to the recommended standard practice of watchful waiting after tick removal, prophylactic antibiotic treatment of tick bites can potentially play an important role in the prevention of Lyme borreliosis[17]. Currently, we are analyzing the data from a randomized controlled intervention study through our tick bite notification website www.tekenradar.nl[18, 19], investigating the efficacy of prophylactic antibiotic treatment after a tick bite within the European setting. With our model, we aimed to identify patients with high risk of developing Lyme borreliosis. Assessment of the duration of the tick’s blood meal and detection of Borrelia DNA in the tick were the best predictors for the risk of developing Lyme borreliosis in our model, with a maximum of 14.4% risk of developing Lyme borreliosis after a single tick bite of a substantially engorged tick that tested positive for Borrelia. Further research is needed to explore how such risk assessment could be implemented for clinical decision making by physicians, since it would involve rapid testing of ticks for infection with Borrelia and estimation of tick attachment duration. To our knowledge, commercially available easy to use and affordable assays for testing of ticks for DNA of Borrelia at point of care currently do not provide suitable sensitivity or specificity for early detection of individuals with a higher risk of Lyme borreliosis.[19] To assess the duration of the tick’s blood meal, specific knowledge and skills of the physicians in general practice would be required. To differentiate between low engorgement (1.4% risk) and substantial engorgement (5.5% risk), physicians training and education would be instrumental. Furthermore, the feasibility of teleconsulting, to send a picture of the tick to a trained entomologist for immediate consultation, could be explored.

## Supporting information

S1 Table Summary measures of the combined dataset of 3,525 single tick bite reports from three prospective tick bite studies. (DOCX)

S2 Table JAGS model code: Structural equation model to quantify the risk of Lyme borreliosis after a tick bite. (DOCX)

S3 Table Probability of developing Lyme borreliosis after a single tick bite, with predictors: Developmental stage of the tick, tick engorgement, tick infection with *Borrelia burgdorferi* s.l. DNA, and patient-estimated duration of tick attachment. (DOCX)

S1 File The combined dataset of 3,525 single tick bite reports from three prospective tick bite studies. (XLSX)
