# Supplementary material for: Predicting the risk of Lyme borreliosis after a tick bite, using a structural equation model
Source: PLoS One. 2017 Jul 24;12(7):e0181807. doi: 10.1371/journal.pone.0181807 (PMC5524385; doi:10.1371/journal.pone.0181807)
Supplement: S1 Table — (DOCX) [file pone.0181807.s001.docx]

**S1 Table. Summary measures of the combined dataset of 3,525 single tick bite reports from three prospective tick bite studies.**

| **Combined dataset** | | | | | | | **TR1213** | | **GP0708** | | **EP0911** | |
| --- | --- | --- | --- | --- | --- | --- | --- | --- | --- | --- | --- | --- |
|  | | | | | N | *(%)* | N | *(%)* | N | *(%)* |  | N |
| **Single tick bite reports** | | | | | 3525 |  | 3021 |  | 260 |  |  | 244 |
| **Lyme borreliosis after a tick bite** | | | | | |  |  |  |  |  |  |  |
|  | no | | | | 1923 | *(97%)* | 1549 | *(97%)* | 206 | *(99%)* | 168 | *(97%)* |
|  | yes | | | | 50 | *(3%)* | 42 | *(3%)* | 3 | *(1%)* | 5 | *(3%)* |
|  | NA | | | | 1552 | *-* | 1430 | - | 51 | - | 71 | *-* |
| **Age of participants** | | | <20 years | | 670 | *(25%)* | 530 | *(24%)* | 29 | *(12%)* | 111 | *(46%)* |
| 20-39 years | | | | | 421 | *(16%)* | 345 | *(16%)* | 33 | *(13%)* | 43 | *(18%)* |
| 40-69 years | | | | | 1400 | *(52%)* | 1194 | *(55%)* | 137 | *(55%)* | 69 | *(29%)* |
| >69 years | | | | | 185 | *(7%)* | 115 | *(5%)* | 51 | *(20%)* | 19 | *(8%)* |
| NA | | | | | 849 | *-* | 837 | - | 10 | - | 2 | *-* |
| Median age (min-max) | | | | | 46 (0-86) | | 46 (0-85) | | 54 (6-86) | | 25 (0-83) | |
| **Developmental stage of *Ixodes ricinus* tick** | | | | | | |  |  |  |  |  |  |
|  | | | larva | | 53 | *(3%)* | 48 | *(3%)* | 2 | *(1%)* | 3 | *(2%)* |
|  |  |  | nymph | | 1341 | *(68%)* | 1113 | *(70%)* | 103 | *(51%)* | 125 | *(67%)* |
|  |  |  | adult | | 573 | *(29%)* | 418 | *(26%)* | 96 | *(48%)* | 59 | *(32%)* |
|  |  |  | NA | | 1558 | - | 1442 | - | 59 | - | 57 | - |
| **Tick engorgement** | | | low | | 802 | *(42%)* | 676 | *(43%)* | 63 | *(33%)* | 63 | *(36%)* |
|  |  |  | moderate | | 856 | *(44%)* | 678 | *(43%)* | 84 | *(44%)* | 94 | *(53%)* |
|  |  |  | substantial | | 270 | *(14%)* | 209 | *(13%)* | 42 | *(22%)* | 19 | *(11%)* |
| NA | | | | | 1597 | *-* | 1458 | - | 71 | *-* | 68 | *-* |
| **Tick infection with *Borrelia burgdorferi* s.l. DNA** | | | | no | 1587 | *(78%)* | 1255 | *(79%)* | 143 | *(69%)* | 189 | *(83%)* |
|  |  |  |  | yes | 439 | *(22%)* | 337 | *(21%)* | 64 | *(31%)* | 38 | *(17%)* |
| NA | | | | | 1499 | *-* | 1429 | - | 53 | - | 17 | *-* |
| **Patient-estimated tick attachment duration** | | | | | | |  |  |  |  |  |  |
| <6 hours | | | | | 582 | *(19%)* | 553 | *(22%)* | 29 | *(12%)* | - | *-* |
| 6 to 12 hours | | | | | 355 | *(12%)* | 336 | *(13%)* | 19 | *(8%)* | - | *-* |
| <12 hours (lower bound NA) | | | | | 130 | *(4%)* | 44 | *(2%)* | - | *-* | 86 | *(36%)* |
| 12 to 24 hours | | | | | 1101 | *(37%)* | 972 | *(39%)* | 59 | *(25%)* | 70 | *(30%)* |
| <24 hours (lower bound NA) | | | | | 9 | *(0%)* | - | *-* | 9 | *(4%)* | - | *-* |
| >24 hours (upper bound NA) | | | | | 83 | *(3%)* | 2 | *(0%)* | - | *-* | 81 | *(34%)* |
| 24 to 48 hours | | | | | 112 | *(4%)* | 60 | *(2%)* | 52 | *(22%)* | - | *-* |
| 48 to 96 hours | | | | | 509 | *(17%)* | 460 | *(18%)* | 49 | *(21%)* | - | *-* |
| 96 to 192 hours | | | | | 92 | *(3%)* | 75 | *(3%)* | 17 | *(7%)* | - | *-* |
| >192 hours | | | | | 15 | *(1%)* | 11 | *(0%)* | 4 | *(2%)* | - | *-* |
| NA | | | | | 537 | *-* | 508 | - | 22 | - | 7 | *-* |
| NA:  TR1213:  GP0708:  EP0911: | | not available  an ongoing web-based national survey www.tekenradar.nl, through which civilians reported tick bites between March 2012 and March 2013.^[19, 20]^  a nationwide prospective study among patients with tick bites who consulted one of 307 enrolling general practitioners in 2007 and 2008.^[13]^  a prospective study performed among patients with tick bites who visited one of fourteen medical emergency posts for consultation of a general practitioner outside office hours from 2009 to 2011. | | | | | | | | | | |
