# Supplementary material for: Predicting the risk of Lyme borreliosis after a tick bite, using a structural equation model
Source: PLoS One. 2017 Jul 24;12(7):e0181807. doi: 10.1371/journal.pone.0181807 (PMC5524385; doi:10.1371/journal.pone.0181807)
Supplement: S2 Table — (DOCX) [file pone.0181807.s002.docx]

**S2 Table.**

**JAGS model code: structural equation model to quantify the risk of Lyme borreliosis after a tick bite.**

model {

# Likelihood

for (i in 1:n) {

# Lyme infection has a Bernoulli distribution

# Probability of infection is a function of log attachment time, tick Borrelia status and engorgement

# cloglog link + linear log attachtment time = Weibull distribution for attachment time

# p = 1 - exp(-lambda*t^nu) -> ... -> log(-log(1 - p)) = cloglog(p) = log(lambda) + nu*log(t), so

# log.lambda[1:3] = baseline hazard or intercept in Weibull function

# nu = nu parameter in Weibull function. Ensure nu > 0

# betaI = log(HR) for Borrelia

# Writing it like this improves MCMC mixing

I[i] ~ dbern(pIres[i])

pIres[i] <- max(0.0001, min(pI[i], 0.9999)) # Restricted pI

cloglog(pI[i]) <- log.lambda[1]*(E[i] == 1) +

log.lambda[2]*(E[i] == 2) +

log.lambda[3]*(E[i] == 3) +

nu*(log.T[i] - mean.log.T) +

betaI*B[i]

# Attachment time has a log-Normal distribution

# Log attachment time is interval censored

# Mean of log attachment time is a function of age

log.T.cat[i] ~ dinterval(log.T[i], log.T.breaks[i, 1:2])

log.T[i] ~ dnorm(muT[i], tauT)

muT[i] <- betaT[1] +

betaT[2]*(Age[i] - mean.Age)

# Tick Borrelia status has a Bernoulli distribution

# Probability of tick Borrelia status is a function of tick stage

# Writing it like this improves MCMC mixing

B[i] ~ dbern(pB[i])

logit(pB[i]) <- betaB[1]*(S[i] == 1) +

betaB[2]*(S[i] == 2) +

betaB[3]*(S[i] == 3)

# Tick engorgement has a categorical distribution

# Probability of tick engorgement is a function of log attachment time and tick stage

# This is a the cumulative logit model

E[i] ~ dcat(pE[i, 1:3])

pE[i, 1] <- cum.pE[i, 1]

pE[i, 2] <- cum.pE[i, 2] - cum.pE[i, 1]

pE[i, 3] <- 1 - cum.pE[i, 2]

logit(cum.pE[i, 1]) <- 0 - muE[i] # First intercept is set to zero here. See betaE[4] below

logit(cum.pE[i, 2]) <- alphaE - muE[i] # Second intercept must be > 0. See prior for alphaE

muE[i] <- betaE[1]*(log.T[i] - mean.log.T) +

betaE[2]*(log.T[i] - mean.log.T)^2 +

betaE[3]*(log.T[i] - mean.log.T)^3 +

betaE[4]*(S[i] == 1) + # First intercept is either betaE[4], betaE[5] or betaE[6]

betaE[5]*(S[i] == 2) +

betaE[6]*(S[i] == 3)

# Tick stage has a categorical distribution

# Probability of tick stage is a function of nothing

S[i] ~ dcat(pS[1:3])

# Age has a categorical distribution

# Probability of age is a function of nothing

Age[i] ~ dcat(pAge[1:nAge])

}

# Priors Lyme infection parameters

for (k in 1:3) {

log.lambda[k] ~ dnorm(0, 0.001)

}

nu ~ dnorm(0, 0.001)I(0, )

betaI ~ dnorm(0, 0.001)

# Priors attachment time parameters

for (k in 1:2) {

betaT[k] ~ dnorm(0, 0.001)

}

tauT ~ dgamma(0.001, 0.001)

# Priors tick Borrelia status parameters

for (k in 1:3) {

betaB[k] ~ dnorm(0, 0.001)

}

# Priors engorgement parameters

for (k in 1:6) {

betaE[k] ~ dnorm(0, 0.001)

}

alphaE ~ dnorm(0, 0.001)I(0, )

# Prior tick stage

pS[1:3] ~ ddirch(alphaS[1:3])

# Prior age

pAge[1:nAge] ~ ddirch(alphaAge[1:nAge])

}
