# Supplementary material for: Predicting the risk of Lyme borreliosis after a tick bite, using a structural equation model
Source: PLoS One. 2017 Jul 24;12(7):e0181807. doi: 10.1371/journal.pone.0181807 (PMC5524385; doi:10.1371/journal.pone.0181807)
Supplement: S3 Table — (DOCX) [file pone.0181807.s003.docx]

**S3 Table.**

**Probability of developing Lyme borreliosis after a single tick bite, with predictors: developmental stage of the tick, tick engorgement, tick infection with *Borrelia* *burgdorferi* s.l. DNA, and patient-estimated duration of tick attachment.**

| **Tick infection with  *B.* *burgdorferi* s.l. DNA** | **Tick stage** | **Engorgement** | **Risk%** | **(95% CI)** | | | | |
| --- | --- | --- | --- | --- | --- | --- | --- | --- |
| unknown | unknown | unknown | 2.6 | ( | 1.4 | - | 5.1 | )  ) |
| unknown | larva | unknown | 2.1 | ( | 1.1 | - | 4.1 | ) |
| unknown | nymph | unknown | 2.5 | ( | 1.4 | - | 4.9 | )  ) |
| unknown | adult | unknown | 2.7 | ( | 1.5 | - | 5.5 | ) |
| unknown | unknown | low | 1.4 | ( | 0.7 | - | 2.3 | )  ) |
| unknown | larva | low | 1.0 | ( | 0.4 | - | 1.7 | ) |
| unknown | nymph | low | 1.3 | ( | 0.6 | - | 2.2 | )  ) |
| unknown | adult | low | 1.6 | ( | 0.8 | - | 2.7 | ) |
| unknown | unknown | moderate | 2.8 | ( | 1.6 | - | 4.2 | )  ) |
| unknown | larva | moderate | 2.0 | ( | 1.1 | - | 3.2 | ) |
| unknown | nymph | moderate | 2.7 | ( | 1.6 | - | 4.0 | )  ) |
| unknown | adult | moderate | 3.2 | ( | 1.9 | - | 4.8 | ) |
| unknown | unknown | substantial | 5.5 | ( | 2.8 | - | 9.2 | )  ) |
| unknown | larva | substantial | 3.9 | ( | 1.8 | - | 6.8 | ) |
| unknown | nymph | substantial | 5.3 | ( | 2.6 | - | 8.7 | )  ) |
| unknown | adult | substantial | 6.4 | ( | 3.2 | - | 10.6 | ) |
| positive | unknown | unknown | 6.7 | ( | 3.6 | - | 13.5 | )  ) |
| positive | larva | unknown | 7.7 | ( | 4.0 | - | 14.9 | ) |
| positive | nymph | unknown | 6.9 | ( | 3.7 | - | 13.7 | )  ) |
| positive | adult | unknown | 6.3 | ( | 3.3 | - | 12.6 | ) |
| positive | unknown | low | 3.6 | ( | 1.7 | - | 6.3 | )  ) |
| positive | larva | low | 3.6 | ( | 1.7 | - | 6.3 | ) |
| positive | nymph | low | 3.6 | ( | 1.7 | - | 6.3 | )  ) |
| positive | adult | low | 3.6 | ( | 1.7 | - | 6.3 | ) |
| positive | unknown | moderate | 7.4 | ( | 4.1 | - | 11.5 | )  ) |
| positive | larva | moderate | 7.4 | ( | 4.1 | - | 11.5 | ) |
| positive | nymph | moderate | 7.4 | ( | 4.1 | - | 11.5 | )  ) |
| positive | adult | moderate | 7.4 | ( | 4.1 | - | 11.5 | ) |
| positive | unknown | substantial | 14.4 | ( | 6.8 | - | 24.6 | )  ) |
| positive | larva | substantial | 14.4 | ( | 6.8 | - | 24.6 | ) |
| positive | nymph | substantial | 14.4 | ( | 6.8 | - | 24.6 | )  ) |
| positive | adult | substantial | 14.4 | ( | 6.8 | - | 24.6 | ) |
| negative | unknown | unknown | 1.4 | ( | 0.7 | - | 2.9 | )  ) |
| negative | larva | unknown | 1.6 | ( | 0.8 | - | 3.2 | ) |
| negative | nymph | unknown | 1.4 | ( | 0.7 | - | 3.0 | )  ) |
| negative | adult | unknown | 1.3 | ( | 0.6 | - | 2.7 | ) |
| negative | unknown | low | 0.7 | ( | 0.3 | - | 1.4 | )  ) |
| negative | larva | low | 0.7 | ( | 0.3 | - | 1.4 | ) |
| negative | nymph | low | 0.7 | ( | 0.3 | - | 1.4 | )  ) |
| negative | adult | low | 0.7 | ( | 0.3 | - | 1.4 | ) |
| negative | unknown | moderate | 1.5 | ( | 0.8 | - | 2.5 | )  ) |
| negative | larva | moderate | 1.5 | ( | 0.8 | - | 2.5 | ) |
| negative | nymph | moderate | 1.5 | ( | 0.8 | - | 2.5 | )  ) |
| negative | adult | moderate | 1.5 | ( | 0.8 | - | 2.5 | ) |
| negative | unknown | substantial | 3.1 | ( | 1.4 | - | 5.6 | )  ) |
| negative | larva | substantial | 3.1 | ( | 1.4 | - | 5.6 | ) |
| negative | nymph | substantial | 3.1 | ( | 1.4 | - | 5.6 | )  ) |
| negative | adult | substantial | 3.1 | ( | 1.4 | - | 5.6 | ) |

| **Tick infection with  *B.* *burgdorferi* s.l. DNA** | **Tick stage** | **Patient-estimated tick attachment (hours)** | **Risk%** | **(95% CI)** | | | | |
| --- | --- | --- | --- | --- | --- | --- | --- | --- |
| unknown | unknown | <12 hours | 2.0 | ( | 1.3 | - | 2.8 | )  ) |
| unknown | unknown | 12 to 24 hours | 2.4 | ( | 1.7 | - | 3.1 | ) |
| unknown | unknown | 24 to 48 hours | 2.8 | ( | 2.1 | - | 3.8 | )  ) |
| unknown | unknown | 2 to 4 days | 3.6 | ( | 2.5 | - | 5.2 | ) |
| unknown | unknown | ≥ 4 days | 5.2 | ( | 3.0 | - | 8.9 | )  ) |
| unknown | larva | <12 hours | 1.7 | ( | 1.0 | - | 2.5 | ) |
| unknown | larva | 12 to 24 hours | 2.0 | ( | 1.3 | - | 2.9 | )  ) |
| unknown | larva | 24 to 48 hours | 2.3 | ( | 1.5 | - | 3.5 | ) |
| unknown | larva | 2 to 4 days | 2.9 | ( | 1.8 | - | 4.5 | )  ) |
| unknown | larva | ≥ 4 days | 3.9 | ( | 2.1 | - | 7.0 | ) |
| unknown | nymph | <12 hours | 2.0 | ( | 1.3 | - | 2.7 | )  ) |
| unknown | nymph | 12 to 24 hours | 2.3 | ( | 1.7 | - | 3.1 | ) |
| unknown | nymph | 24 to 48 hours | 2.8 | ( | 2.0 | - | 3.7 | )  ) |
| unknown | nymph | 2 to 4 days | 3.5 | ( | 2.4 | - | 5.1 | ) |
| unknown | nymph | ≥ 4 days | 5.0 | ( | 2.9 | - | 8.6 | )  ) |
| unknown | adult | <12 hours | 2.2 | ( | 1.4 | - | 3.0 | ) |
| unknown | adult | 12 to 24 hours | 2.6 | ( | 1.8 | - | 3.4 | )  ) |
| unknown | adult | 24 to 48 hours | 3.0 | ( | 2.2 | - | 4.1 | ) |
| unknown | adult | 2 to 4 days | 3.9 | ( | 2.6 | - | 5.6 | )  ) |
| unknown | adult | ≥ 4 days | 5.7 | ( | 3.3 | - | 9.9 | ) |
| positive | unknown | <12 hours | 5.4 | ( | 3.2 | - | 7.8 | )  ) |
| positive | unknown | 12 to 24 hours | 6.3 | ( | 4.2 | - | 8.9 | ) |
| positive | unknown | 24 to 48 hours | 7.5 | ( | 4.9 | - | 10.7 | )  ) |
| positive | unknown | 2 to 4 days | 9.5 | ( | 5.9 | - | 14.3 | ) |
| positive | unknown | ≥ 4 days | 13.5 | ( | 7.4 | - | 23.5 | )  ) |
| positive | larva | <12 hours | 6.2 | ( | 3.6 | - | 9.2 | ) |
| positive | larva | 12 to 24 hours | 7.3 | ( | 4.7 | - | 10.5 | )  ) |
| positive | larva | 24 to 48 hours | 8.6 | ( | 5.5 | - | 12.7 | ) |
| positive | larva | 2 to 4 days | 10.7 | ( | 6.6 | - | 16.6 | )  ) |
| positive | larva | ≥ 4 days | 14.4 | ( | 7.7 | - | 24.5 | ) |
| positive | nymph | <12 hours | 5.5 | ( | 3.3 | - | 8.0 | )  ) |
| positive | nymph | 12 to 24 hours | 6.5 | ( | 4.3 | - | 9.1 | ) |
| positive | nymph | 24 to 48 hours | 7.7 | ( | 5.0 | - | 11.0 | )  ) |
| positive | nymph | 2 to 4 days | 9.7 | ( | 6.1 | - | 14.7 | ) |
| positive | nymph | ≥ 4 days | 13.7 | ( | 7.5 | - | 23.8 | )  ) |
| positive | adult | <12 hours | 5.0 | ( | 3.0 | - | 7.3 | ) |
| positive | adult | 12 to 24 hours | 5.9 | ( | 3.8 | - | 8.3 | )  ) |
| positive | adult | 24 to 48 hours | 7.0 | ( | 4.6 | - | 9.9 | ) |
| positive | adult | 2 to 4 days | 8.8 | ( | 5.6 | - | 13.3 | )  ) |
| positive | adult | ≥ 4 days | 12.9 | ( | 7.1 | - | 23.0 | ) |
| negative | unknown | <12 hours | 1.1 | ( | 0.6 | - | 1.7 | )  ) |
| negative | unknown | 12 to 24 hours | 1.3 | ( | 0.8 | - | 1.9 | ) |
| negative | unknown | 24 to 48 hours | 1.6 | ( | 1.0 | - | 2.3 | )  ) |
| negative | unknown | 2 to 4 days | 2.0 | ( | 1.2 | - | 3.1 | ) |
| negative | unknown | ≥ 4 days | 2.9 | ( | 1.5 | - | 5.4 | )  ) |
| negative | larva | <12 hours | 1.3 | ( | 0.7 | - | 2.0 | ) |
| negative | larva | 12 to 24 hours | 1.5 | ( | 0.9 | - | 2.3 | )  ) |
| negative | larva | 24 to 48 hours | 1.8 | ( | 1.1 | - | 2.7 | ) |
| negative | larva | 2 to 4 days | 2.3 | ( | 1.3 | - | 3.6 | )  ) |
| negative | larva | ≥ 4 days | 3.1 | ( | 1.6 | - | 5.6 | ) |
| negative | nymph | <12 hours | 1.1 | ( | 0.6 | - | 1.7 | )  ) |
| negative | nymph | 12 to 24 hours | 1.4 | ( | 0.8 | - | 2.0 | ) |
| negative | nymph | 24 to 48 hours | 1.6 | ( | 1.0 | - | 2.4 | )  ) |
| negative | nymph | 2 to 4 days | 2.1 | ( | 1.2 | - | 3.2 | ) |
| negative | nymph | ≥ 4 days | 3.0 | ( | 1.5 | - | 5.4 | )  ) |
| negative | adult | <12 hours | 1.0 | ( | 0.6 | - | 1.6 | ) |
| negative | adult | 12 to 24 hours | 1.2 | ( | 0.7 | - | 1.8 | )  ) |
| negative | adult | 24 to 48 hours | 1.5 | ( | 0.9 | - | 2.2 | ) |
| negative | adult | 2 to 4 days | 1.9 | ( | 1.1 | - | 2.9 | )  ) |
| negative | adult | ≥ 4 days | 2.8 | ( | 1.4 | - | 5.3 | ) |
